# Formulation of a Composite Nasal Spray Enabling Enhanced Surface Coverage and Prophylaxis of SARS‐COV‐2

**DOI:** 10.1002/adma.202008304

**Published:** 2021-05-31

**Authors:** Richard J. A. Moakes, Scott P. Davies, Zania Stamataki, Liam M. Grover

**Affiliations:** ^1^ Healthcare Technology Institute School of Chemical Engineering University of Birmingham Birmingham B15 2TT UK; ^2^ Institute of Immunology and Immunotherapy School of Medicine and Dentistry University of Birmingham Birmingham B15 2GW UK

**Keywords:** carrageenan, COVID‐19, formulation engineering, nasal sprays, SARS‐CoV‐2

## Abstract

Airborne pathogens pose high risks in terms of both contraction and transmission within the respiratory pathways, particularly the nasal region. However, there is little in the way of adequate intervention that can protect an individual or prevent further spread. This study reports on a nasal formulation with the capacity to combat such challenges, focusing on severe acute respiratory syndrome coronavirus‐2 (SARS‐CoV‐2). Formulation of a polysaccharide‐based spray, known for its mucoadhesive properties, is undertaken and it is characterized for its mechanical, spray distribution, and antiviral properties. The ability to engineer key mechanical characteristics such as dynamic yield stresses and high coverage is shown, through systematic understanding of the composite mixture containing both gellan and λ‐carrageenan. Furthermore, the spray systems demonstrate highly potent capacities to prevent SARS‐CoV‐2 infection in Vero cells, resulting in complete inhibition when either treating, the cells, or the virus, prior to challenging for infection. From this data, a mechanism for both prophylaxis and prevention is proposed; where entrapment within a polymeric coating sterically blocks virus uptake into the cells, inactivating the virus, and allowing clearance within the viscous medium. As such, a fully preventative spray is formulated, targeted at protecting the lining of the upper respiratory pathways against SARS‐CoV‐2.

## Graphical abstract

There is a high risk of transmission of airborne pathogens through the nasal epithelium. This study reports a novel nasal spray, which is designed to maximize surface coverage and prevent infection through physical entrapment and inhibition of infection by severe acute respiratory syndrome coronavirus‐2 (SARS‐CoV‐2).

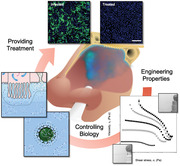

## 1 Introduction

There are many airborne viruses including: influenza‐, rhino‐, adreno‐, entero‐, and coronavirus. The latter, coronaviridae (CoVs) family, are implicated in a variety of gastrointestinal, central nervous system, and respiratory diseases (Middle East Respiratory Syndrome (MERS), severe acute respiratory syndrome (SARS);^[^ ^1^ ^]^ with the latest strain, severe acute respiratory syndrome coronavirus‐2 (SARS‐CoV‐2), receiving much attention due to its devastating impact within the 2020 pandemic. SARS‐CoV‐2, like all coronaviruses, contains large positive‐strand RNA genomes packed within a helical capsid, all housed within a phospholipid bilayer envelope formed on budding.^[^ ^2^, ^3^ ^]^ Associated with the viral membrane are 3 main proteins: membrane and envelope proteins, associated with assembly, and spike proteins. The spike proteins, which give rise to its corona shape, are essential for virus survival, mediating entry to the host cell.^[^ ^4^, ^5^ ^]^ Additionally, the protein also plays a crucial role in determining host range and tissue tropism, alongside being responsible for inducing many of the host immune responses.^[^ ^1^ ^]^ To date, facilitation of viral entry into a host cell is believed to arise through specific motifs within the spike protein, which strongly interact with Angiotensin‐Converting Enzyme 2 (ACE2) receptors.^[^ ^6^, ^7^ ^]^ ACE2 is known for its role in regulating oxygen/carbon dioxide transfer, commonly found within the respiratory epithelia. In particular, SARS‐CoV‐2 has been found to target the ciliated and goblet cells,^[^ ^8^ ^]^ where subsequent viral shedding results in extensive viral loads, especially within the upper respiratory tract.^[^ ^9^ ^]^

Inhaled air is primarily routed through the nose. Even though the nasal passages present the highest resistance to airflow, on average ≈10 000 L of air are inhaled by a healthy human per day.^[^ ^10^, ^11^ ^]^ Only once this pathway becomes overloaded does the body switch to respiration through the mouth.^[^ ^12^, ^13^ ^]^ For this reason, the nasal cavity supports two major roles: air conditioning, creating the correct levels of humidity and air temperature; and, removal of foreign particles including dust, airborne droplets and pathogens.^[^ ^14^ ^]^ Anatomically, the nose consists of two cavities roughly 10 cm in length and half again in height, producing a total surface area of about 150 cm^2^.^[^ ^15^ ^]^ Inspired air flows up through the nasal vestibule (nostril) and passes through the slit‐like meatus structures (inferior, middle, and superior) and back through the nasopharynx. At a cellular level, the majority of the cavity consists of a typical airway epithelium, comprising of four main cell types: basal, ciliated/non‐ciliated columnar, and goblet cells. The columnar cells, whether ciliated or not, are coated by microvilli. Their role, to prevent drying, supports the cilia in performing mucociliary clearance of mucin‐rich fluids secreted by the goblet cells.^[^ ^16^, ^17^ ^]^ Additionally, the presence of cilia and microvilli drastically increases the effective surface area (≈9.6 m^2^), providing a highly efficient platform for filtration.^[^ ^18^ ^]^ Unfortunately, such large surface areas also provide greater exposure in terms of viral entry.

The airborne risk imposed not only through ventilation systems and crowds but re‐suspension of the virus from inanimate objects, including personal protective equipment,^[^ ^19^ ^]^ emphasizes the need for new and novel devices that not only prevent contraction but stop spread thereafter. Several advances within the nasal spray field have sought to address similar challenges, drawing on various technological approaches. Many of these attempts can be crudely categorized into two main areas: active targeting of the virus (e.g., products such as SaNOtize) and passively protecting the mucosa from viral uptake (e.g., Taffix, Vicks First Defence). The former, albeit by far an inexhaustive list, has seen the incorporation of novel drug molecules being delivered intranasally,^[^ ^20^ ^]^ and translation of antimicrobial research focused on reactive species;^[^ ^21^, ^22^ ^]^ for example, reactive oxygen and nitric oxide is used to directly target SARS‐CoV‐2.^[^ ^23^, ^24^ ^]^ Although such products show great potential, their mechanisms of action, constantly bordering on pharmacological, significantly slow their movement from lab to clinic. A more physical approach, by creating a passive barrier to viral uptake, presents a much quicker route. As such products are typically based on viscosity modifiers, for example, hydroxypropyl methylcellulose or carrageenan, aiming to enhance retention and slow diffusion of the virus across the mucosa. Indeed, more recent sprays have focused on spraying dry powders, which in the presence of limited fluids in the nasal cavity, form a highly concentrated polymer layer with gel‐like characteristics.^[^ ^25^ ^]^ It is important to note, that although these have been described here as passive, as a secondary role, such products often provide an acidic environment reported to reduce viral activity.^[^ ^26^ ^]^ Although many of these products can be effective, they are often compromised through poor user compliance, where a tendency to jet from the packaging results in both poor coverage and irritation.^[^ ^27^, ^28^ ^]^

This study looks to address the challenges faced by nasal sprays by engineering high‐viscosity materials with apparent yielding behaviors (ensuring maximal retention in the nasal cavity), the ability to actively target virus removal through entrapment and maintain high surface coverage: all of which are required to provide adequate protection and consumer compliance. The emphasis on speed within such unprecedented times, in terms of translating the fundamental science from lab to clinic, drives key considerations such as simplicity and proven biocompatibility. Therefore, colloidal composites of Food and Drug Administration (FDA)‐approved polymers were studied for their ease of translation and known chemical attributes such as mucoadhesion; delivering not only a system with the potential to move to clinic but enhance protection through adhesion to the nasal mucosa. Systems were deconstructed back to their single constituents, and characterized for their mechanical, spray, and antiviral properties. As such a set of design principles was determined, based on a structure function relationship, in order to present a potential nasal spray to combat airborne pathogens, in particular SARS‐CoV‐2.

## 2 Results

### 2.1 Physico‐Mechanical Behaviors of the Nasal Spray Formulation

**Figure 1:**
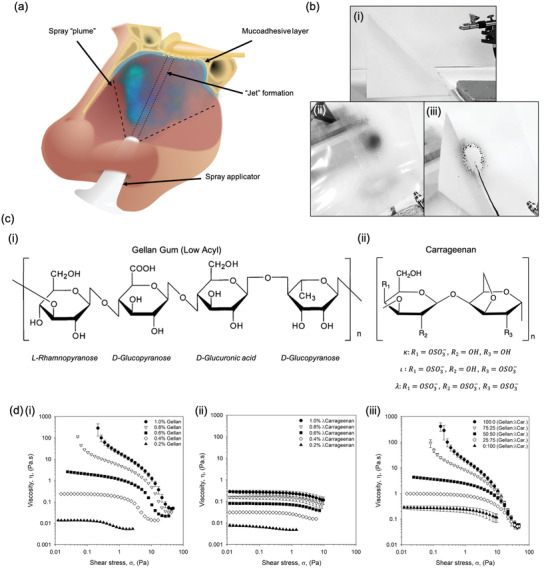
Defined nasal spray behaviors. a) Schematic diagram demonstrating the application of a nasal spray to the nasal cavity. b) Typical images obtained during screening of numerous mucoadhesive polymers for their ability to evenly spray and be retained on a 45° incline: i) spray set up, ii) gellan gum 1% (w/v) with black dye, and iii) alginate 1% (w/v) with black dye. c) Molecular structures of: i) gellan gum (low acyl), and; ii) carrageenan, where changes in the “R” groups provide variations for *k*, ι, and *l*. d) Dynamic viscosity profiles from high to low shear stress for: i) gellan samples with concentrations ranging from 0.2 to 1.0% (w/v), ii) *l*‐carrageenan samples with concentrations ranging from 0.2 to 1.0% (w/v), and iii) composite systems of gellan:*l*‐carrageenan at a total polymer concentration of 1% (w/v).

On application, nasal sprays directly contact the nasal mucosa lining the epithelium (**Figure** **1**a). Nasal residence time can be improved via careful choice of the polymer, promoting interaction with the mucus; known as mucoadhesion. As such, a range of polymers known for their mucoadhesive properties (gellan, carrageenan, alginate, pectin, dextran), were dispersed in dilute phosphate‐buffered saline (providing a buffering capacity against their native acid/basic pH, without substantially altering the solvent quality) and studied for their potential as a nasal spray. Narrowing of these initial candidates was achieved by screening their ability to form a homogenous sprayed layer. In addition, although not directly simulating a nasal mucosa, the ability to self‐support upon a 45° incline was also a determining factor as to the acceptability of the polymer within the starting formulation—demonstrating the ability to prevent the unpleasant “running” sensation when applied (Figure 1b). Figure 1b‐ii,iii shows typical images for several of the polymers tested, demonstrating a “good” and “poor” candidate; gellan and alginate respectively. Screening in this manner provided a means to narrow the systems down to both gellan and carrageenan (chemical structures shown in Figure 1c), with others either creating heterogeneous distributions or flowing under their own mass.

Flow behaviors were characterized via dynamic viscosity (from high to low shear stress), representative of the material once sprayed. Resultant profiles for the gellan were modeled demonstrating a transition from power law to Cross model, suggesting the loss of dynamic yield stress to zero‐shear viscosity as a function of the polymer concentration (Figure 1d‐i). No transition was observed for the λ‐carrageenan systems, characterized solely by the Cross model at all polymer concentrations studied (Figure 1). Zero‐shear viscosity was dependent on polymer content, providing viscosities within the range of 0.27 to 0.01 mPa.s for 1.0 to 0.2% (w/v), respectively.

Viscosity curves for the composite mixtures containing both the gellan and the λ‐carrageenan (ratios of 100:0, 75:25, 50:50, 25:75, and 0:100) have been shown in Figure 1d(iii) and **Table** **1**. Flow behaviors for the 1% (w/v) systems showed a clear transition from material characteristics indicative of the gellan (viscosity asymptoting at low stresses), to those of the λ‐carrageenan (plateaued viscosities at low stresses), as the ratio of the two polymers shifted from one extreme to the other (gellan to λ‐carrageenan). Loss of overall viscosity was also observed as the systems shifted from high to low gellan ratios, confirmed by the reduction in consistency coefficient (*K*) from 3.54 to 0.03. This correlated well with the increase in rate index (*n*), where more gellan resulted in higher degrees of shear‐thinning: 0.40 to 0.82 for 100% gellan and 100% λ‐carrageenan, respectively. A reduction in the total polymer content to 0.4% (w/v) resulted in all mixtures characterized by the Cross model, consistent with data provided for the isolated polymers. Further reduction in the polymer concentration, to 0.2% (w/v), resulted in profiles independent of the ratio of gellan to λ‐carrageenan, with samples indistinguishable from each other (within error).

**Table 1:** Comparison of viscometry data. Tabulated viscometry data compiled for composite systems modeled either using the power law model (no zero‐shear data provided) or Cross model (zero‐shear data) (error values show the 95% confidence interval)

**Total Polymer [% (*w*/*v*)]**	**Polymer ratio (gellan:*l*‐carrageenan)**	**Zero‐shear viscosity [Pa s]**	**Consistency coefficient (*K*)**	**Rate index (*n*)**
1.0	100:0	N/a	3.544 ± 0.319	0.403 ± 0.004
	75:25	N/a	2.693 ± 0.075	0.491 ± 0.002
	50:50	4.080 ± 0.324	1.163 ± 0.034	0.543 ± 0.005
	25:75	0.988 ± 0.013	0.094 ± 0.002	0.727 ± 0.012
	0:100	0.274 ± 0.046	0.030 ± 0.009	0.821 ± 0.151
0.4	100:0	0.245 ± 0.002	0.065 ± 0.004	0.831 ± 0.003
	75:25	0.172 ± 0.005	0.060 ± 0.001	0.692 ± 0.054
	50:50	0.083 ± 0.001	0.020 ± 0.002	1.021 ± 0.084
	25:75	0.052 ± 0.002	0.016 ± 0.001	1.134 ± 0.029
	0:100	0.032 ± 0.001	0.013 ± 0.001	1.293 ± 0.051
0.2	100:0	0.014 ± 0.001	0.021 ± 0.001	1.315 ± 0.161
	75:25	0.009 ± 0.001	0.022 ± 0.001	1.413 ± 0.025
	50:50	0.010 ± 0.001	0.017 ± 0.003	1.341 ± 0.287
	25:75	0.007 ± 0.001	0.016 ± 0.007	1.283 ± 0.403
	0:100	0.007 ± 0.001	0.026 ± 0.001	1.288 ± 0.056

Viscometry data was used to better understand the potential residence of the spray within the nasal cavity. As such, Equation (1) was used to predict the stress exerted on the material under gravity residing on an incline.

(1) $$\begin{array}{c} \sigma_{\max}=\rho gh(\sin\theta ) \end{array}$$

where ρ is the density of the nasal spray (kg m^−3^), *g* is the force due to gravity (9.807 m.s^−2^), *h* is the thickness of the sprayed layer (*m*), and θ is the inclined angle. Applying values for the polymer suspensions based on a maximum 100 μm thick sprayed layer at 45° (Equation (2)) resulted in a theoretical stress of 0.7 Pa.

(2) $$\begin{array}{c} \sigma_{\max}=(1010)\;\times \;(9.807)\;\times \;(1\;\times \;10^{-4})\left(\sin\left(45\right)\right) \end{array}$$

A simple force balance revealed insufficient stress under gravity to induce flow in any of the systems containing dynamic yield stress. Indeed, even in systems described by the Cross model, the external stress due to gravity was not sufficient to move the system from its zero‐shear plateau into the thinning region.

### 2.2 Understanding Formulation Sprayability

**Figure 2:**
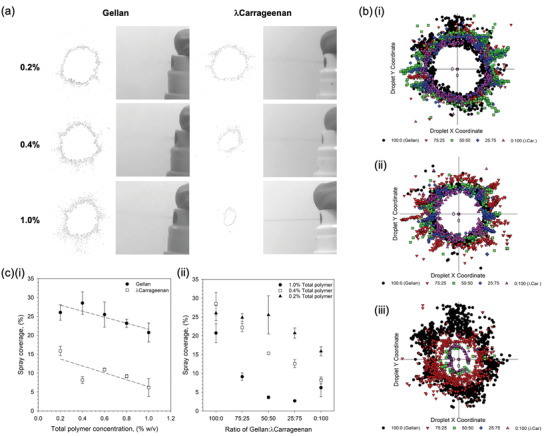
Sprayability of polymer suspensions. a) typical images of the spray formation as the polymer suspensions are aspirated form the applicator, alongside resulting distribution outlines for a range of polymer concentrations. Rapid loss of spray acuity moving away from the aperture for gellan systems is a result of the “plume” formation. b) overlay of droplet distributions from a central point showing the reduction in spray as a function of the ratio of gellan to *l*‐carrageenan for: i) 0.2% (w/v) total polymer, ii) 0.4% (w/v) total polymer, and iii) 1% (w/v) total polymer. c) Spray coverage, as determined using an imaging software, for: i) single polymer suspensions (trend lines are denoted by the dashed line with *R*2 values of 0.72 and 0.62 for the gellan and *l*‐carrageenan, respectively), and ii) composite mixtures of the gellan and *l*‐carrageenan at either 0.2, 0.4, or 1.0% (w/v) total polymer.

In order to compare spray distributions as a function of the physico‐chemical properties and not the applicator, whilst maintaining forces representative of manual pumping, a simple manual spray pump was used: the more intricate chambers found within nasal pumps can prevent homogenous flow of more viscous materials, providing non‐comparable results. Application of the polymeric materials has been shown in **Figure** **2**. Spray distributions for the single polymer systems have been demonstrated in Figure 2a. Gellan demonstrated an inherent ability to spray forming a typical “plume” across all concentrations studied: where wider distribution of the material resulted in rapid loss of acuity, as the density of the material quickly reduced further from the pump aperture. In contrast, even at the lowest concentration, λ‐carrageenan systems demonstrated a degree of “jetting”, becoming more visible as the polymer concentration increased. Adoption of either a “plume” or “jet” on being expelled from the applicator was reflected in the distributions formed on contact with the substrate (Figure 2b). Here, following increasing polymer, distributions became narrower with fewer satellite droplets forming around the central accumulation. A general negative correlation between %coverage and total polymer concentration was drawn, loosely fitting a linear trend (*R* ^2^ = 0.72 and 0.62 for both gellan and λ‐carrageenan, respectively) (Figure 2c‐i). Furthermore, it was observed that all gellan concentrations resulted in higher coverage than the λ‐carrageenan, demonstrating maximum and minimum %coverage of 28.5–20.7% when compared to 15.9–6.1% for the λ‐carrageenan systems (0.2 and 1.0% (w/v) polymer, respectively).

The role that overall and ratio of polymers play within the sprayability of the composite systems can be clearly seen in Figure 2b. In all instances, irrespective of total polymer concentration, a shift to smaller distributions was observed as the ratio of gellan to λ‐carrageen decreased. Such changes became more pronounced with total polymer, where the magnitude of change between 100% gellan to 100% λ‐carrageenan, followed 1.0% > 0.4% > 0.2% (w/v). such observations were mirrored in the total coverage data (Figure 2c(ii)). Replacing 25% of the total λ‐carrageenan with gellan resulted in a 4.9% and 4.4% increase in coverage, for the 0.2% and 0.4% (w/v) systems; with an initial loss in spray coverage (−3.5%) for the 1% total polymer content. Coverage was further increased to 9.0%, 14.1%, and 2.9% for the 0.2, 0.4, and 1.0% (w/v) systems respectively at a ratio of 75:25 (gellan:λ‐carrageenan).

### 2.3 In Vitro Inhibition of SARS‐CoV‐2

**Figure 3:**
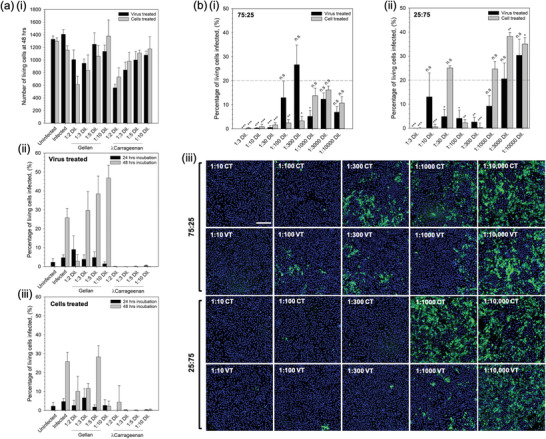
First hit and transmission analysis prevention. a) In vitro SARS‐CoV‐2 assay using vero cells to determine: i) cell tolerance to the nasal sprays (live/dead analysis), ii) degree of infection at 24 and 48 h for cells inoculated with the virus having undergone a pre‐treatment with either the gellan or *l*‐carrageenan spray, and iii) degree of infection at 24 and 48 h for spray‐treated cells inoculated with the virus. b) Degree of infection for the composite mixtures (1% (w/v) total polymer) after 48 h incubation having undergone either the virus treated or cell treated regimens for: i) 75:25% gellan to *l*‐carrageenan, or ii) 25:75% gellan to *l*‐carrageenan systems (the dotted line shows the mean value for the non‐treated control), and iii) typical fluorescence microscopy images of treated systems using Hoechst staining; scale bar: 200 μm (blue denotes non‐infected and green infected cells). (n.s.: not statistically different, *:*p* < 0.05, **:*p* < 0.01, and ***:*p* < 0.001)

First hit and ability to prevent person to person transmission of the virus was studied in vitro using SARS‐CoV‐2 infection of Vero cells. An initial study was undertaken to determine cell viability when exposed to the sprays over a 48 h incubation period (**Figure** 3a‐i). Cell tolerance was dependent on the polymer concentration, demonstrating a twofold reduction in the number of living cells for both the gellan and the λ‐carrageenan at a dilution of 1/2. Dose‐response of cell viability was linear (*R* ^2^ = 0.96 and 0.97 for gellan and λ‐carrageenan, respectively), with reduced cell death as the systems became increasing more dilute.

Prevention of both contraction and/or transmission of the virus was assessed by two treatment regimens: treating the virus with the compound prior to infecting the cells (referred to as virus treated); or, by first treating the cells before introduction of the virus (referred to as cells treated).

Figure 3a‐ii,iii show the effect of the single polymer systems on resultant infection when treated with the virus‐ and cell‐first regimens, respectively. It was observed that in the case of the gellan only, all dilutions resulted in infection irrespective of treatment regime after 24 h. Indeed after 48 h, such observations were even clearer with dilutions greater than 1/3 resulting in levels of infection above the control. Interestingly, the λ‐carrageenan treated systems showed no signs of infection above the uninfected control at either time point, 24 or 48 h, irrespective of the treatment regimen.

Composite systems containing 1% total polymer at either a ratio of 75:25 or 25:75 (gellan to λ‐carrageenan) were also studied using the same treatment regimens over 48 h; data presented in Figure 3b. Composites of a ratio 75:25 showed significant suppression of the infection (minimum of *p* < 0.05) up to a dilution of 1/100 on comparison with the untreated control group (Figure 3b‐i). In contrast, composites at a ratio of 25:75 comprising a higher proportion of λ‐carrageenan, demonstrated fluctuations in suppression with dilutions of 1/30, 1/1000, 1/3000, and 1/10 000 all resulting in infection levels equal to or greater than the untreated control (Figure 3b‐ii). Comparison of the treatment regimens highlighted key differences in the ability to suppress infection. Again, for the 25:75 composite, it can be seen that at lower dilution factors, between 1/3 and 1/300 (with the exception of 1/30), resulted in lower average responses for cells treated prior to infection when compared to treating the virus first. However, at larger dilution factors (>1/300) it became apparent that treatment of the virus first becomes more effective. This can be seen more clearly in the images showing Hoechst‐stained cells, where the extent of infected cells (green: spike 2 protein staining) was much less for the virus treated groups when compared to the cell treated groups.

### 2.4 Spray Mechanism of Inhibition

**Figure 4:**
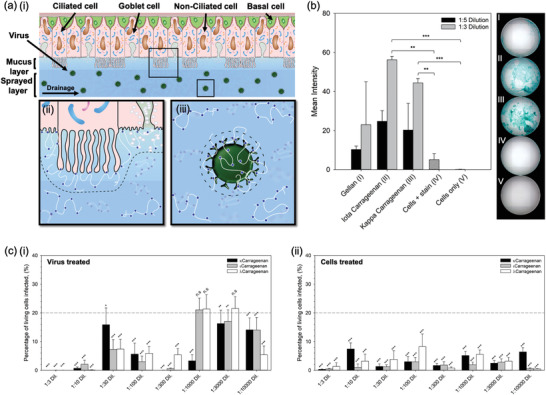
Mechanism for the inhibition of SARS‐CoV‐2. a) Schematic diagram showing the nasal epithelium covered in the nasal spray: i) demonstration of potential removal of the virus via trapping within the sprayed layer and elimination through natural nasal clearance mechanisms (sneezing/nose‐blowing/swallowing), ii) demonstration of potential blockage of virus uptake into the cells as the polymer creates a steric barrier across the cell interface, and iii) demonstration of potential inhibition of virus uptake by creating a steric barrier around the interface of the virus. b) Alcian blue stain intensity for cells treated and subsequently washed with either gellan, ι‐carrageenan or *l*‐carrageenan. c) In vitro SARS‐CoV‐2 assay using Vero cells to determine levels of infection after 48 h for systems treated with increasingly sulphated carrageenans (*k* < ι < *l*), by either: i) pre‐treating the virus, or ii) pre‐treating the cells (the dotted line shows the mean value for the non‐treated control, with statistical significance being a pair‐wise comparison to this data). (n.s.: not statistically different, *:*p* < 0.05, **:*p* < 0.01, and ***:*p* < 0.001)

The influence of polymer and degree of sulphation was studied in order to better understand the mechanism of infection inhibition. Initial experiments were conducted to ascertain adherence of the polymer to the cell membrane. Staining (Alcian blue) of the sugar chains was conducted post‐treatment and washing. **Figure** **4**b shows staining intensity as a function of: polymer type, gellan and carrageenan; and, degree of sulphation along the carrageenan backbone, ι and λ.

Intensity data highlighted a significant difference (*p* < 0.001) between cells treated with a 1/3 dilution of both carrageenans when compared to the cells only group. Moreover, when compared to the stained cells only group, significance remained (*p* < 0.01). Inter‐carrageenan analysis demonstrated ι‐carrageenan to have higher average intensity in comparison to the λ‐carrageenan (56.2% and 44.4%, respectively). To determine whether the degree of sulphation across the polymer backbone was important in suppression of the infection, κ‐, ι‐, and λ‐carrageenan were studied using the SARS‐CoV‐2 assay (Figure 4c). It was observed that in all cases, where the cells were treated prior to being exposed to the virus, infection was lowered to below the untreated control group (*p* < 0.001). This could not be said for the pre‐treated virus, where larger dilution factors (1/1000 and 1/3000) did not statistically affect the degree of infection for both the ι‐ and λ‐carrageenans. Additionally, no correlation could be drawn to the extent of sulphation and its ability to suppress infection.

## 3 Discussion

The role that the nasal passage plays in frontline defense, filtering harmful bacteria and viruses, naturally elevates the sinonasal pathways to high risk, in terms of infection.^[^ ^29^ ^]^ The need to formulate medicines/devices which can help regulate and protect this area is thus clear, however, like many regions of the body the nasal cavity poses many challenges, due to: ease of access, dynamics (native clearing mechanism), and topology (inclined surfaces or ceilings). As such, formulation engineering plays a decisive role in the design of novel therapeutics.^[^ ^30^ ^]^ The link between microstructure and material properties has long been known, ultimately driving macroscopic responses key to both function (delivery/retention/ADME – absorption, distribution, metabolism and elimination) and the end user (ability to administer/patient compliance). Through a microstructural design approach, the interplay between areas such as raw materials and processing can be manipulated to engineer defined characteristics. In the case of a nasal spray elements such as mucoadhesion, longevity, coverage, and controlled delivery/prophylaxis need to be considered. The use of polysaccharides within biological applications are becoming increasingly more frequent due to their often biocompatible nature, with many having been approved by regulatory bodies such as the FDA for use in pharmaceuticals; significantly reducing risk, time, and costs throughout the translational process.

In addition to biocompatibility and widespread regulatory approval, both gellan and carrageenan are known to demonstrate intrinsic mucoadhesive properties. Ultimately, the ability to adhere to the mucosa is governed through interactions with mucins in the mucus. The mucus blanket within the nasal mucosa composes of two layers; the periciliary liquid covered by a gel‐like structure.^[^ ^31^ ^]^ Although comprising of ≈90% water, nasal mucus gains its viscoelastic structure through mucins, highly branched, high molecular weight *O‐*glycoproteins, which are both anchored across the epithelium and distributed extracellularly.^[^ ^32^ ^]^ Adhesion of the spray is facilitated through physical entanglement, driven by the long polymeric chains (high molecular weights, >100 kDa),^[^ ^33^, ^34^, ^35^ ^]^ augmented through ionic interactions with charged side groups (—COO^−^, —SO_3_^−^) and van der Waals forces.^[^ ^36^, ^37^ ^]^ This results in high retention on the mucosa and a mechanism of clearance, becoming transported by the cilia out of the paranasal sinuses to the pharynx and eventually into the oesophagus.^[^ ^16^, ^17^ ^]^

Enhancing longevity within the nasal cavity can also be achieved by increasing the spray's viscosity, resulting in reduced flow/clearance. The role that gellan and carrageenan play within viscosity modification and related sensory attributes have been well established within the food industry.^[^ ^38^ ^]^ Again, owing to their long polymeric chains and chemistries along their backbone, both gellan and λ‐carrageenan are able to structure large volumes of water. This, accompanying polymer‐polymer entanglements, ultimately drives increases in viscosity,^[^ ^39^, ^40^ ^]^ with higher polymer concentrations resulting in more viscous suspensions: until sufficiently concentrated in the case of the gellan (>0.8% (w/v)), providing the evolution of dynamic yield stress. Again, yielding behavior can be used to enhance application and slow clearance, as the gravitational stress is insufficient to cause rupture of the film formed post‐spraying; where the film height can be estimated as a function of a typical nasal dosage (25–200 μL)^[^ ^41^ ^]^ over a surface area ≈5 cm^2^.^[^ ^18^ ^]^

The large surface areas in the nasal cavity provide the ability to process large volumes of air (up to 35 L min^−1^ before switching to oronasal breathing), within a total volume of ≈15 mL.^[^ ^18^, ^41^ ^]^ However, the large nasal area presents a challenge to uniformly coat. Coverage of the polymer systems demonstrated clear correlations between both the type of polymer and the concentration of polymer used. Gellan systems demonstrated high levels of coverage across all concentrations studied, suggesting an ideal candidate for nasal spray application. Interestingly, λ‐carrageenan even though characterized by a lower viscosity, resulted in poor overall coverage whilst still maintaining concentration dependency. Such changes were a direct result of a shift from plume to jet formation, with gellan resulting in much faster rates of jet destabilization in comparison to the λ‐carrageenan. Spray behaviors comply with literature, suggesting that large surface tensions, as opposed to viscosity, are required to force droplet breakup, relative to the density of the surrounding medium.^[^ ^42^ ^]^ As a result, the persistence of a jet negatively affects patient compliance, not only providing poor coverage but eliciting unwarranted irritation on contact with the nasal wall.^[^ ^27^, ^43^ ^]^

To maintain the advantage of λ‐carrageenan's intrinsic anti‐viral capacity,^[^ ^44^, ^45^, ^46^, ^47^, ^48^, ^49^, ^50^, ^51^ ^]^ formulation of a composite mixture containing increasing amounts of gellan to λ‐carrageenan allowed for optimization of the nasal therapy. Careful control over the two polymers provided a means to engineer enhanced λ‐carrageenan sprayability. Interchanging 25% of the initial λ‐carrageenan with gellan saw an increase in the total area coated up to ≈35% of its initial coverage. This was further increased to ≈63% on the replacement of 75% of the initial polymer. In addition to tailorable spray profiles, composite systems demonstrated a means to formulate sprays containing λ‐carrageenan with both yielding and augmented viscosities, not possible with the λ‐carrageenan alone. Data showed that the formation of intermediate products, from 100% λ‐carrageenan to 100% gellan, transitioned in behavior governed primarily by the dominating polymer. As such, it was possible to detail a set of design principles that can be used to formulate various sprays, with desired mechanical properties.

Cytotoxicity and anti‐viral activity of the nasal treatments were assessed using a relevant enveloped virus, SARS‐CoV‐2, and their current gold‐standard model for infection (Vero cells). Initial cytotoxicity studies revealed a degree of cell death when cultured in the presence of both the gellan and λ‐carrageenan. The abundance of literature demonstrating the compatibility and use of such polysaccharides in pharma and biomaterials^[^ ^52^ ^]^ might suggest that such observations are indeed an artifact of 2D cell culture, as opposed to inherent toxicity. It is thought that the simplified nature of cell culture does not account for the complex transport phenomena and underlying tissues which would usually support overlying cells, making cells more robust within in vivo situations. In addition, the high sugar concentrations coupled with relatively low ionic species (as a result of only 5% (v) PBS) at low dilution factors, may also affect the equilibrium in tonicity, resulting in osmotic stress and cell shock/death.^[^ ^53^, ^54^ ^]^ Again, this demonstrates the disparity between 2D culture and an in vivo setting, where the complex high ionic environment of the nasal mucus would help shift the equilibrium back towards an isotonic nature.^[^ ^31^ ^]^ However, the potential role that dead cells/cellular debris could play in terms of interference, within the in vitro results, with regard to viral adhesion/infection at such low dilutions cannot be excluded.

First hit and ability to inactivate the virus is passed from infected to non‐infected patient (prevent viral transmission) was assessed using two treatment regimens; treatment of cells prior to infection, and, pre‐treatment of the virus, respectively. Firstly, prophylaxis was assessed through application of the spray onto the cells prior to infection. Gellan systems showed limited ability to suppress the SARS‐CoV‐2 virus, whereas, λ‐carrageenan demonstrated complete inhibition over 48 h. Pre‐treatment of the virus to assess ascertain whether it remained infectious post‐treatment, similarly reduced infection, demonstrating complete inhibition; supporting previous acknowledgements that λ‐carrageenan provides enhanced anti‐viral capacities. Composites again provided the ability to accommodate synergistic behaviors from both gellan and λ‐carrageenan: enhanced mechanical responses towards spraying and anti‐viral activity. Indeed, the spray was highly potent with dose‐dependency demonstrating significant prevention/reduction of infection up to 30‐ and 300‐fold dilutions for the virus and cell treatments, respectively. Interestingly, systems containing a greater proportion of gellan outperformed the λ‐carrageenan dominated system; an unexpected outcome based on the single polymer data. It is hypothesized that such observations are driven through the distribution of electrostatic charge carried by the carrageenan within the solvent, enabling binding to the cellular membrane. It has previously been reported that sugars, within ternary (sugar‐salt‐water) solutions, have a significant effect in structuring the water molecules.^[^ ^55^, ^56^ ^]^ It is considered that when gellan gum is at higher ratio (once highly diluted), it facilitates this role, leaving the carrageenan less hindered. In addition, reduced ion mobility, lowering ionic screening, promotes membrane protein–carrageenan interactions, which may explain why the polymer blend has a more potent anti‐viral effect.^[^ ^57^ ^]^ However, systematic measurements of the interactions within these systems are required to prove this: falling outside the scope of this manuscript.

Inhibition of the infection is thought to take place through 3 main mechanisms: formation of a steric barrier at the cell interface, adsorption of the polymer to the virus, and/or physical entrapment of the virus in the sprayed layer. It is proposed that polymer adsorption is facilitated through charge–charge interactions at the cell and virus membrane. Although both anionic in nature, the contrast in virus inhibition infers that the carrageenan's sulphate chemistry drives anchoring of the polymer to the substrate surface. The polymer thus provides a physical role, expanding the hydrodynamic volume around the cell/virus and preventing close proximity.^[^ ^58^ ^]^ Even though the role that the negatively charged sulphate groups play in the ability to adsorb to the bio‐interface, it is unclear from the data whether a link between the degree of sulphation and suppression of infection exists. Although not significant in the role of coating, gellan does demonstrate its applicability when considering prophylaxis through entrapment and elimination, augmenting the protection afforded by the already available mucin barrier. The ability to engineer high viscosities and yielding behavior at this point becomes key, proportionally slowing diffusivity, as described by the Stokes–Einstein relation.^[^ ^59^ ^]^ To this end, diffusion of the virus towards the host cells can be hampered within timescales associated with typical nasal clearance.^[^ ^60^ ^]^ In reality, a combination of the three proposed mechanisms are likely to be in operation (Figure 4a), requiring a more clinically relevant model to fully demonstrate the spray's antiviral action; potentially facilitated by the natural production of a mucus layer, through culture of epithelia cells at an air–liquid interface.^[^ ^61^, ^62^, ^63^ ^]^ As such, physical entrapment is suggested to provide a first means of defense, simultaneously resulting in a secondary defense where cells and virus become coated. Thus, any virus particles having migrated to the cell interface are already inhibited to uptake. This combinatorial approach, coupled with the highly potent anti‐viral capacity of the carrageenan towards SARS‐CoV‐2, provides a powerful spray device with the capacity to prevent both contraction and transmission.

## 4 Conclusions

As the primary mode of transmission for airborne viruses is uptake through the respiratory tract, the nasal passage poses one of the largest risk factors to contraction. Although it is well known that the nose filters thousands of liters of air daily, there is little in the way of preventative measures to ensure protection against infection. This study has demonstrated the formulation of a potent antiviral nasal spray, with not only prophylactic capacity, but the ability to prevent viral transmission. Its ability to completely inhibit infection is derived from the chemistry (sulphated polymer backbone) of the active polymer, λ‐carrageenan. Spray characteristics were engineered through the production of a composite, where a set of design rules were understood to allow for manipulation over the material behaviors: spray coverage, viscosity, and yielding behavior. Furthermore, understanding the role of each polymer in the composite allowed for a preventative mechanism, using the synergy of both material and antiviral properties to coat the biological interfaces, prevent viral uptake by host cells, and eliminate through native clearance pathways. As such, this work presents a potential device with the capacity to specifically target infection within the nasal cavity.

## 5 Experimental Section

### Materials

Sodium alginate (medium viscosity) (pro.#: A2033; Lot.#: SLBZ2709), pectin from citrus peel (pro.#: P9135; Lot.#: SLBH9128v), κ‐carrageenan (pro.#: 22048; Lot.# BCBX5072), ι‐carrageenan (pro.#: C1138; Lot.#: SLBT4542), *l*‐carrageenan (pro.#: 22049; Lot.#: BCBP8978v), PBS, heat inactivated FBS, penicillin/streptomycin, Alcian blue (8GX) were all purchased from Sigma Life Science, UK; dextran (Mw ≈20 kDa) (pro.#: J61216); Lot.#: U11D023) was purchased from Alfa Aesar; gellan gum (CG‐LA) was purchased from CP Kelco; TrypLE Express 1x was purchased from Fisher Scientific; black dye (Parker); Type‐1 water (Milli‐Q, Merck Millipore).

### Preparation of Single‐Component Systems

Colloidal suspensions were prepared through the addition of polymer (0.2 to 1.0% (w/v)) to a dilute PBS (5% v) solution. Once added, the systems were vigorously mixed and left to fully hydrate for 24 h. All samples were kept at ambient temperature (≈20 °C) until further used.

### Preparation of Multi‐Component Systems

Composite mixtures were prepared by first weighing out ratios of polymer (75:25, 50:50, 25:75—gellan gum (low acyl) to *l*‐carrageenan), and thoroughly mixing. Powdered mixtures (0.2, 0.4, and 1.0% (w/v) total polymer concentration) were then added to a dilute PBS (5% v) solution, vigorously mixed and left to fully hydrate for 24 h. All samples were kept at ambient temperature (≈20 °C) until further use.

### Polymer Screening

Polymer screening was conducted using an airbrush (750 mm aperture) coupled to an oil‐free compressor (Badger, USA), set to 1 bar. The test material (0.9 mL) was mixed with black dye (0.1 mL) and sprayed across an acetate sheet set to a 45° incline. The airbrush was then cleaned using a succession of 70% ethanol and water. Spray distributions were visually analyzed for homogeneity and retention.

### Rheological Characterization

Viscometric analysis was undertaken on a rotational rheometer (Kinexus Ultra, Netzsch Geratebeu GmbH, DE) fitted with a cone and plate (4°, 40 mm diameter) geometry. Tests were conducted at 25 °C, under stress control. Dynamic viscosity was analyzed by reduction of the shear stress from a maximum of 100 to 0.001 Pa (dependent on test material to prevent expulsion from the gap at lower viscosities) over a 2 min ramp time. Kinexus software was used to characterize the flow profiles using both power‐law and Cross models.

### Sprayability

The test material was first mixed with black dye (0.1% v) and thoroughly shaken to provide a homogenous mixture. A typical handheld applicator (Adelphi, UK) was used to vertically spray a paper recipient. Sprayed distributions were allowed to dry in air (no blotting effects observed) and scanned at 600 DPI (grayscale). Image files were processed using an image package (ImageJ), where they were initially cropped to a 2000 by 2000 px box visually centered around the spray pattern. Standard thresholding was applied to all images, and scale corrected equating 2000 px to 100%. Droplet analysis was conducted, and total coverage determined as a percentage of the whole image. Distributions were recorded as *x*/*y* coordinates and plotted relative to the central droplet.

### Infection/Transmission Analysis

The Vero cells were washed with PBS, dislodged with 0.25% Trypsin–EDTA (Sigma life sciences), and seeded into 96‐well imaging plates (Greiner) at a density of 10^4^ cells per well in culture media (Dulbecco's modified Eagle medium (DMEM) containing 10% FBS, 1% penicillin and streptomycin, 1% l‐glutamine and 1% non‐essential amino acids). Cells were incubated for 24 h to allow time for adherence. Virus or cells were treated with polymeric solutions, diluted in media, 1 h prior to infections. Cells were subsequently infected with SARS‐CoV‐2 virus England 2 stock 10^6^ IUml^−1^ (kind gift from Christine Bruce, Public Health England) diluted 1/150 in culture media. Cells were fixed in ice‐cold MeOH after infection. Cells were then washed in PBS and stained with rabbit anti‐SARS‐CoV‐2 spike protein, subunit 1 (The Native Antigen Company), followed by Alexa Fluor 555‐conjugated goat anti‐rabbit IgG secondary antibody (Invitrogen, Thermofisher). Cell nuclei were visualized with Hoechst 33342 (Thermofisher). Cells were washed with PBS and then imaged and analyzed using a ThermoScientific CellInsight CX5 High‐Content Screening (HCS) platform. Infected cells were scored by perinuclear fluorescence above a set threshold determined by positive (untreated) and negative (uninfected) controls.

### Cell Binding Studies

The Vero cells were expanded in T75 flasks, washed with PBS (5 mL) and removed using TrypLE (2.5 mL). The cells were then re‐suspended in complete media and seeded into 96 well plates (10 000 cells per well). Cells were left to attach over the subsequent 24 h prior to treatment. Cells were washed (3 times) with PBS and final washing removed. Test material was diluted to either 1/3 or 1/5 and placed over the cells (200 μL) (controls were treated with equal volumes of PBS). Cells were incubated for 30 min prior to washing (3 times) with PBS. Cells were subsequently stained with Alcian blue (0.1%) for 30 min, before a final wash in PBS to remove residual stain. PBS was then added (200 mL) and wells imaged. Cells were imaged using a Cytation 5M automated microplate imager. Wells were imaged in bright field using a 4× optical lens focused on the center of each well. Wells were divided into a 6 × 4 matrix and stitched together retrospectively. Images were then cropped to the well diameter using a software package (ImageJ) and color thresholding standardized and analyzed for mean intensity.

### Statistical Analysis

In all experiments, data presented were an average of at least triplicates, with error portrayed as the 95% confidence interval. Significance was determined by first assessing data for normality. Where normally distributed, paired *t*‐tests were conducted comparing the treatment group to the untreated control. If the normality test failed, comparison was made on ranks using the Mann–Whitney test. Significance has been shown on plots using the following notation: n.s – not statistically different; *: *p* < 0.05; **: *p* < 0.01; and, ***: *p* < 0.001.

## Conflict of Interest

The authors declare no conflict of interest.

## Data Availability

Research data are not shared.
